# IFN-τ Maintains Immune Tolerance by Promoting M2 Macrophage Polarization via Modulation of Bta-miR-30b-5p in Early Uterine Pregnancy in Dairy Cows

**DOI:** 10.3390/cells14020087

**Published:** 2025-01-10

**Authors:** Xinyu Feng, Cheng Yang, Ting Wang, Jinxin Zhang, Han Zhou, Bin Ma, Ming Xu, Ganzhen Deng

**Affiliations:** Department of Clinical Veterinary Medicine, College of Veterinary Medicine, Huazhong Agricultural University, Wuhan 430070, China; fengxinyu666777@webmail.hzau.edu.cn (X.F.); cheng.yang@webmail.hzau.edu.cn (C.Y.); wt2000@webmail.hzau.edu.cn (T.W.); zhangjinxin@webmail.hzau.edu.cn (J.Z.); zhouhan@webmail.hzau.edu.cn (H.Z.); 2021302110210@webmail.hzau.edu.cn (B.M.)

**Keywords:** IFN-τ, SOCS1, pregnancy, M2 macrophage, bta-miR-30b-5p

## Abstract

Pregnancy failure in the first trimester of cows significantly impacts the efficiency of the dairy industry. As a type I interferon exclusively to ruminants, IFN-τ plays a key role in maternal recognition and immune tolerance of fetuses. Macrophages are the most common immune cells within the ruminant endometrium. Nevertheless, deeply analyzing the mechanisms of IFN-τ regulating macrophage polarization still needs further study. In this study, a notable decline of bta-miR-30b-5p expression via the increase of SOCS1 was observed in uterine tissues of pregnant cows. We then confirmed that the 3′UTR of SOCS1 was to be directly targeted by bta-miR-30b-5p. After that, we demonstrated that this obviously promoted the bovine macrophages (BoMac) polarized to M2 through enhancing SOCS1 expression with the treatment of IFN-τ. Furthermore, we found that SOCS1 restrained the expression of the key proteins p65 and p-P65 in the NF-κB pathway. Causing, the wide range of cross-species activities of IFN-τ, therefore we established a pregnant mouse model for the future confirmation of the above mechanism. The results verified that IFN-τ significantly improved this mechanism and maintained normal pregnancy status in mice, but miR-30b-5p significantly reduced the M2 polarization by inhibiting SOCS1, which activated the NF-κB signaling pathway, and then leading to the failure of embryo implantation. All these results indicated that IFN-τ can regulate immune tolerance during pregnancy by promoting M2 macrophage polarization through inhibiting bta-miR-30b-5p targeting SOCS1 to deactivate the NF-κB signaling pathway.

## 1. Introduction

Failed gestation causes a low birth rate and therefore extremely decreases the profit of the dairy industry. Statistical data indicate that the fertilization rate of cows is 85~95%, while the birth rate of fetuses is only 30~50% [1,2]. Reproductive issues in cows result in extended calving intervals and hinder reproductive efficiency, which ultimately leads to significant economic losses [3,4]. Addressing reproductive issues in cows occupies a central position within the field of bovine research, and a multitude of studies have been undertaken in this area in the past [5,6,7]. Fetus loss in the first trimester is mainly caused by the failure of fetus implantation, which is usually due to the fetus’ fertility, uterine nutrients, and immune condition [8]. The majority of pregnancy failures in cows occur during the period of maternal recognition of the embryo [9]. In the field of reproductive immunology, the avoidance of excessive immune responses in the early stages of pregnancy represents a significant challenge for researchers.

As a type I interferon exclusively secreted by embryonic trophoblast ectodermal cells in the early stages of pregnancy, IFN-τ plays a key role in maternal–fetal recognition and immune tolerance [10]. IFN-τ prevents luteolysis and maintains progesterone secretion by inhibiting the pulses of 13,14-dihydro-15-keto-PGF2α (PGFM) through silencing estrogen and oxytocin receptors to maintain normal gestation [11,12]. The maternal–fetal immune response is mainly caused by semi-allogeneic proteins, and an extreme response usually induces implantation failure [13,14]. It has been demonstrated that IFN-τ balances the inflammatory and anti-inflammatory condition by regulating uterine immune response, which is a profile to fetus implantation [15,16]. Macrophages are the most proliferating immune cells in the uterus of pregnant cows [17,18]. Macrophages are differentiated into classically activated macrophages (M1) and alternatively activated macrophages (M2) [19]. A balance of polarization is essential for a healthy pregnancy state [20,21]. It has been demonstrated that IFN-τ regulates macrophage polarization to modulate the immune response [22,23]. However, how IFN-τ regulates the immune mechanisms of macrophages in dairy cows remains unknown.

MicroRNAs (miRNAs) exhibit species conservation and engage in extensive and intricate regulatory functions within organisms [24]. The function of miRNAs in the regulation of pregnancy has been the subject of extensive research [25,26,27]. The expression of miRNAs in early pregnancy tissues may be a critical factor in the successful implantation of the embryo, and differential miRNA expression ultimately results in different pregnancy outcomes [28]. Furthermore, it was demonstrated that miRNAs can regulate macrophage polarization [29,30]. For example, miR-21 has been demonstrated to facilitate the phagocytosis of apoptotic cells, thereby enabling a transition from the M1 to the M2 macrophage phenotype [31]. Similarly, miR-33 has been shown to promote macrophage transition to the M2 phenotype by targeting AMP-activated protein kinase [32]. Therefore, the primary purpose of this research was to identify candidate miRNAs that may regulate macrophage proliferation and influence the immune status of pregnancy.

Our previous study collected uterine tissue from cows in the early stages of pregnancy. The subsequent analysis revealed an increase in M2 macrophages in the endometrium. The early stage of bovine pregnancy is a distinctive period of IFN-τ secretion. In light of these findings, we propose the hypothesis that the secretion of IFN-τ in early bovine pregnancy may regulate the maternal immune response through specific molecular mechanisms, thereby ensuring the successful implantation of the embryos. Thus, this experiment aimed to investigate the potential association between IFN-τ and the observed increase in M2-type macrophages in the maternal endometrium following pregnancy. Additionally, the specific mechanism of action of IFN-τ in maintaining initial pregnancy through immune macrophages was examined.

## 2. Materials and Methods

### 2.1. Collection of Bovine Uterine Samples

Bovine uterine tissues were collected from a commercial slaughterhouse in Wuhan, China. Bovines with a corpus luteum in the ovaries and a placenta were evaluated as pregnant, and as non-pregnant without a corpus luteum and placenta. Bovines with a placenta *φ* ≤ 1 cm were defined as in the early pregnant stage. Eight uteri were collected in both the pregnant and non-pregnant group (*n* = 8), respectively. Three uterine cavity tissue samples were collected from each bovine, and washed with sterile PBS, and then transported back to the laboratory for the detection of SCOS1, CD68, CD86, and CD206 with immunohistochemical staining; meanwhile, the SOCS1, TGF-β1, CD206, CCL17, bta-miR-30b-5p levels were analyzed with qRT-PCR.

### 2.2. Cell Culture

The bovine macrophage cell line (BoMac) and HEK-293 were cultured in RPMI1640 complete medium (HyClone, Logan, UT, USA) with 10% bovine fetal serum (FBS, AUSGENEX, Molendinar, QLD, Australia) under 5% CO_2_ at 37 °C. Cells were successively passaged when the density reached 70~90%.

### 2.3. Cell Transfection and Processing

We obtained bta-miR-30b-5p mimics, bta-miR-30b-5p inhibitor, small interfering RNA (si-SOCS1), and the corresponding negative control (NC) from GenePharma (Shanghai, China), and their sequences are presented in Table 1. After the cell density reached 60%, si-SOCS1, bta-miR-30b-5p mimics and inhibitor, and their NC were transfected into BoMac cells using Lipofectamine 2000 (Invitrogen, Waltham, MA, USA). The cells were incubated at 37 °C with 5% CO₂ for 12 h and then treated with 200 ng/mL IFN-τ (Cloud Clone, Wuhan, China) for a further 12 h.

### 2.4. Cell Viability

The viability of cells was determined through the utilization of the Cell Counting Kit (Yeasen, Shanghai, China). To this end, 4 × 10^4^ BoMac cells per well were transferred into 96-well plates and were incubated for 1 h to ensure that the cells adequately adhered to the wall. The fresh culture medium was served as the control, and cells were cultured with the treatment of 200 ng/mL IFN-τ for 0, 6, 12, and 24 h. Subsequently, CCK-8 solution was added to the wells and incubated with 5% CO₂ at 37 °C for 4 h. Absorbance values were quantified using a microplate reader (Bio-Rad, Hercules, CA, USA) at a wavelength of 450 nm.

Cell viability was calculated using the following formula: Cell viability (%) = [A(dosing)—A(blank)]/[A (0 dosing)—A(blank)] × 100, where A (0 dosing) represents the absorbance of wells with cells, CCK-8 solution, and no drug solution.

### 2.5. Immunohistochemistry (IHC)

The uterine tissues were embedded in paraffin and cut into 4-μm-thick sections, and incubated overnight at 4 °C with the primary antibodies of SOCS1 (A7754, ABclonal, Wuhan, China), CD68 (A13286, ABclonal, Wuhan, China), CD86 (A2353, ABclonal, Wuhan, China) and CD206 (18704-1-AP, Proteintech, Wuhan, China), and then incubated with biotinylated goat anti-rabbit mouse secondary antibodies for 2 h. The color was developed using the DAB detection kit, and hematoxylin was used as a counterstain. All histologic pictures were taken and interpreted using a light microscope (Nikon, model E100, Tokyo, Japan).

### 2.6. Immunofluorescence (IF)

Following the fixation of paraffin sections of uterine tissue or cultured cells with 4% paraformaldehyde, a 10-min permeabilization process was conducted using 0.01% Triton X-100 at 37 °C. This was followed by the addition of 10% goat serum. Following the incubation of the primary antibody, the secondary antibody, (Goat Anti-Rabbit Mouse IgG-HRP, M21003S, Abmart, Shanghai, China) was incubated for one hour.

### 2.7. Quantitative Polymerase Chain Reaction (qPCR) Analysis

Total RNA was extracted from tissues and cells with TRIzol Reagent (Invitrogen, Carlsbad, CA, USA), and the concentration and purity of the extracted RNA were assessed by optical density (OD) values at 260/280 nm. Bta-miR-30b-5p level was analyzed by qRT-PCR with miRNA 1st Strand cDNA Synthesis Kit (Stem-loop) (Vazyme, Nanjing, China), and mRNA levels were analyzed by qRT-PCR with HiScript^®^ II Q Select RT SuperMix for qPCR (Vazyme, Nanjing, China). The primer sequences utilized for miRNA and mRNA analysis are presented in Table 2. The comparative 2^−ΔΔCt^ method was employed to quantify the relative expression levels, and the respective reference genes were U6 or GADPH.

### 2.8. Western Blotting

Tissues and cells were lysed using RIPA buffer (Thermo Scientific, Waltham, MA, USA), and their proteins were extracted and determined using a BCA kit (Thermo Scientific, Waltham, MA, USA). Proteins (30 mg) separated by 10% polyacrylamide gel electrophoresis (SDS-PAGE), were transferred to polyvinylidene difluoride (PVDF) membranes and enclosed with 5% skimmed milk powder for 2 h. The above proteins were respectively incubated overnight at 4 °C with the antibody of SOCS1 (A7754), NF-κB p65 (A2547), Phospho-NF-κB p65 (AP1294), CD206 (A8301), Arg1 (A1847), β-Actin (AC006) (all from ABclonal, Wuhan, China) and CD206 (18704-1-AP, Proteintech, Wuhan, China), which was washed and incubated for future use with Goat Anti-Rabbit Mouse IgG-HRP for 2 h, and finally quantified using fully automated chemiluminescence imaging analysis system.

### 2.9. Dual Luciferase Reporter Assay

The targeting site of bta-miR-30b-5p to SOCS1 mRNA 3′UTR was predicted using the TargetScan website. To validate their targeting relationship, bovine wild-type (WT) and mutant SOCS1 mRNA 3′UTR genes were designed amplified, and cloned into the psiCHECK^TM^-2 Vector (Promega, Madison, WI, USA). Subsequently, wild-type and mutant plasmids were co-transfected into HEK293 T cells containing bta-miR-30b-5p mimics or NC. The fluorescence intensity of cell was detected by the Dual-Luciferase^®^ reporter gene analysis system (Promega, Madison, WI, USA) using the Dual-Luciferase Reporter Gene Assay Kit (Promega, Madison, WI, USA), and was used to analyze their targeting relationship.

### 2.10. Animal Experiments

Kunming (KM) mice at the age of 6–8 weeks (from the Laboratory Animal Service Center of Huazhong Agricultural University) were raised with consistent diets and water at 23–25 °C and relative humidity of 40–80%, and with 12-h lighting.

To investigate the impact of bta-miR-30b-5p on the uterine immune environment and its underlying mechanisms during early pregnancy a mouse pregnancy model was established, and controlled with non-pregnant mice. Gestation was confirmed by the investigation of the vaginal plug in every morning and evening. The appearance of the vaginal plug was recorded as day 0.5 of gestation. On days 0.5, 4.5, and 7.5, pregnant mice were divided into five groups (*n* = 5): nonpregnant (Blank), pregnant (NC), pregnant trial 1 (agomiR-NC+PBS), pregnant trial 2 (8 ng/g IFN-τ for one mouse), and pregnant trial 3 (8 ng/g IFN-τ + 5 mg/kg AgomiR-30b-5p for one mouse). IFN-τ or any of the above reagents were administered via intrauterine injection. On day 10.5, the mice were euthanized, and the uteri were collected to observe morphological changes and count the embryos. All animal experiments were conducted following the animal experiment policy of Huazhong Agricultural University (HZAUMO-2015-12).

### 2.11. Statistical Analysis

The mean ± SEM of three physiologically independent experiments or samples are used to represent all quantitative data. Šidák’s multiple comparisons test was applied with one/two-way ANOVA on ranks for multiple comparisons. Statistical analyses were performed using GraphPad Prism 6 (GraphPad Software, Inc., La Jolla, CA, USA). All data were considered statistically significant at * *p* < 0.05, ** *p* < 0.01.

## 3. Results

### 3.1. Recruitment and Bias to the M2 of Macrophages in the Bovine Endometrium in Early Pregnancy and Increased SOCS1 Expression

**Figure 1:**
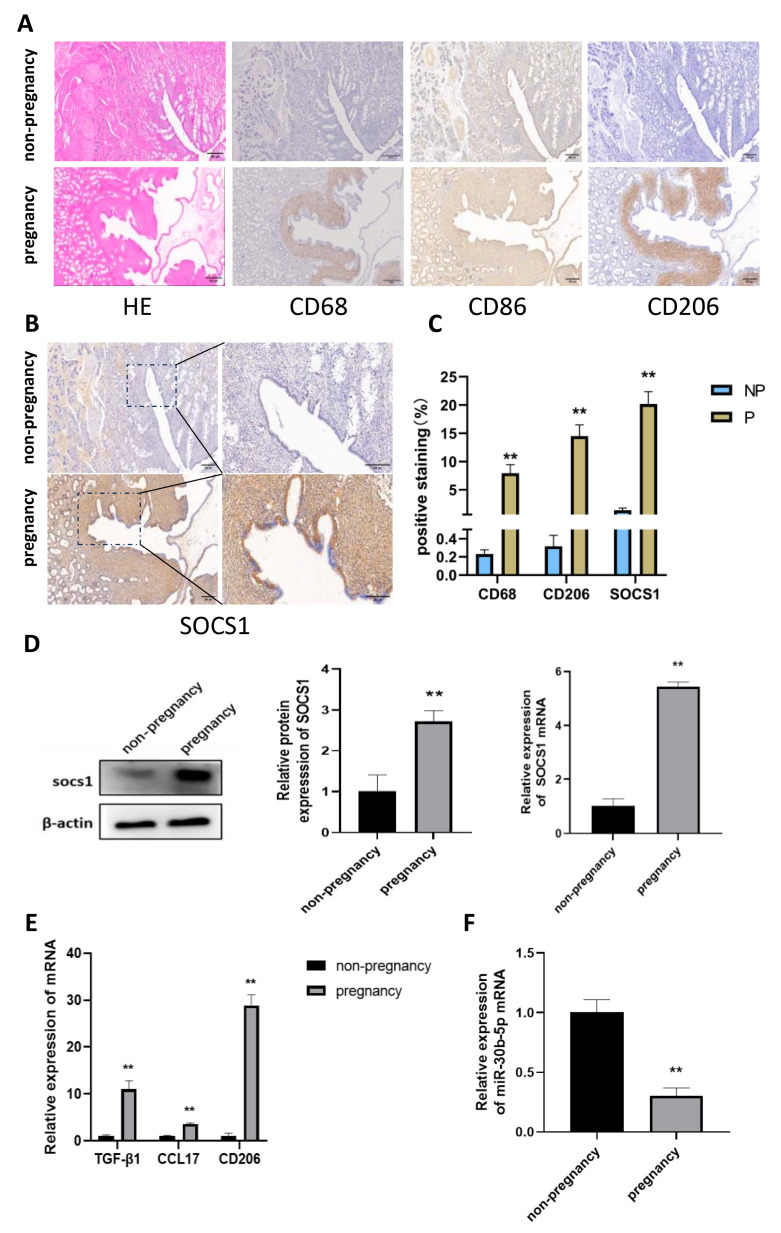
Increased expression of M2 macrophages and SOCS1 and decreased bta-miR-30b-5p in the endometrium of early pregnant dairy cows (**A**) Immunohistochemical staining of CD68, CD86, and CD206 in the endometrium of cows at the beginning of pregnancy (*n* = 8) and non-pregnant cows (*n* = 8) with hematoxylin and eosin to demonstrate the pathological alterations in bovine endometrial tissues (scale bar 500 μm). (**B**) Immunohistochemical staining of SOCS1 at the endometrium of cows at the beginning of pregnancy (*n* = 8) and non-pregnant cows (*n* = 8) (scale bar 500 μm, magnification scale bar 200 μm). (**C**) The gray scale values of CD68, CD206, and SOCS1 protein expression were quantified using the ImageJ software (ImageJ V1.8.0.112). (**D**) SOCS1 expression levels in the endometrium of pregnant (*n* = 8) and non-pregnant cows (*n* = 8) were determined by western blotting and quantitative real-time polymerase chain reaction (qRT-PCR). (**E**) Quantitative reverse transcription polymerase chain reaction (qRT-PCR) analysis of the endometrium of pregnant (*n* = 8) and non-pregnant cows (*n* = 8) was conducted to determine the expression of CD206, CCL17, and TGF-β1 in the endometrium. (**F**) The expression levels of bta-miR-30b-5p in the endometrium of pregnant (*n* = 8) and non-pregnant (*n* = 8) cows were determined. The data were derived from three independent experiments and are expressed as the mean ± SEM. ** *p* < 0.01 (Student’s *t*-test).

It has been established that in ruminants after pregnancy, a large number of macrophages are recruited in the endometrium and become the primary immune cell population. Consequently, an immunohistochemical assay was conducted on the uterine samples obtained from the cows to ascertain alterations in macrophage populations within the collected uterine tissues. The selected markers were CD68, CD86, and CD206. The results are presented in Figure 1A. The number of macrophages at the endometrium was low (*p* < 0.01) in the non-pregnant group, whereas the overall percentage of macrophages (CD68+) increased (*p* < 0.01) in early pregnancy. This confirms that macrophages are recruited to the endometrium in large numbers in early pregnancy. Furthermore, the results revealed an increase (*p* < 0.01) in the number of CD206-positive cells, indicative of M2 macrophages, while the CD86-positive cell population remained unaltered. This indicates that the proportion of M2 macrophages increases significantly in the endometrium following pregnancy. Staining for the SOCS1 protein revealed a substantial region of elevated SOCS1 expression (*p* < 0.01) in the uterine tissues of pregnant cows. Notably, SOCS1 exhibited heightened expression (*p* < 0.01) at the endometrium, where macrophages were concentrated (Figure 1B). Additionally, qRT-PCR and western blot experiments were conducted on the collected uterine tissue samples to detect the mRNA expression and protein level of SOCS1. As illustrated in Figure 1D, the data revealed an increase (*p* < 0.01) in SOCS1 expression when compared to the non-pregnant group. Furthermore, qRT-PCR of M2 macrophage-associated immune factors in tissues demonstrated that the expression of TGF-β1, CCL17, and CD206 immunosuppressive factors in the endometrium of pregnant cows was higher (*p* < 0.01) than that in the non-pregnant group (Figure 1E). In conclusion, these findings indicate that the elevated population of M2 macrophages in the endometrium of pregnant cows may be regulated by the high expression of SOCS1.

### 3.2. Bta-miR-30b-5p Is Negatively Correlated with SOCS1 Expression in Uterine Tissues of Pregnant Cows

The SOCS1 has been found to have high expression in pregnant uterine tissues. To further explore the direction of upstream regulation, miRNAs have been identified as a potential area of interest. The distinctive temporal function of IFN-τ, secreted by embryonic trophoblast ectodermal cells during the early stages of pregnancy in ruminants, has prompted a sustained investigation into its role in this process. In a previous study, our laboratory investigated the differential expression of miRNAs in response to IFN-τ by stimulating bovine endometrial luminal epithelial cells (bEECs) with IFN-τ for RNA sequencing. Based on the sequencing results, and using MiRWalk and TargetScan to screen for miRNAs with potential roles from the overlap, it was found that bta-miR-30b-5p expression was significantly reduced, and there may be a target binding with SOCS1. The reduction of bta-miR-30b-5p suggests that it may play a role in the process of pregnancy. The results of qRT-PCR are shown in Figure 1F. Bta-miR-30b-5p expression was lower (*p* < 0.01) in the endometrium of cows in early pregnancy compared to the non-pregnant group. Given the significant recruitment of macrophages to the endometrium during early pregnancy, it was postulated that endogenous bta-miR-30b-5p expression in these cells may also undergo downregulation.

### 3.3. IFN-τ Treatment of BoMac Downregulates bta-miR-30b-5p and Upregulates SOCS1

**Figure 2:**
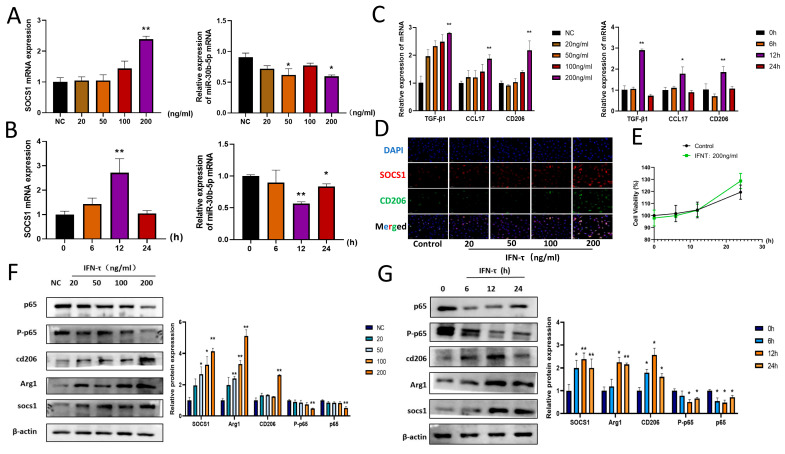
**IFN-τ affects bta-miR-30b-5p, SOCS1, NF-κB signaling pathway and induces M2 polarization** (**A**) BoMac was treated with 20, 50, 100, or 200 ng/mL interferon-τ for 12 h, and the expression of SOCS1 and microRNA-30b-5p was detected by quantitative reverse transcription-polymerase chain reaction (qRT-PCR). (**B**) The expression of SOCS1 and bta-miR-30b-5p was quantified by qRT-PCR following the treatment of BoMac with 200 ng/mL IFN-τ for 0, 6, 12, or 24 h. (**C**) The expression of M2 macrophage-associated immunosuppressive factors was detected by qRT-PCR under different concentration or time conditions. (**D**) Immunofluorescence images of SOCS1 and CD206 (scale bar 50 μm) were obtained after treatment of BoMac with 20, 50, 100, or 200 ng/mL IFN-τ for 12 h. The immunofluorescence images are presented in the following table. (**E**) BoMac was treated with IFN-τ (200 ng/mL) for 0, 6, 12, or 24 h, and cell viability was assessed using the CCK-8 assay. (**F**) The expression levels of target proteins in each group under different concentrations were detected by western blot. (**G**) The expression levels of target proteins in each group under different time conditions were detected by western blot. The data presented here were derived from three independent experiments and are expressed as the mean ± SEM. * *p* < 0.05, ** *p* < 0.01 (Student’s *t*-test).

In order to elucidate the mechanism of SOCS1 and bta-miR-30b-5p in endometrium-recruited macrophages following the action of IFN-τ on the endometrium during the early stages of pregnancy, a cell model was established comprising different IFN-τ stimulation conditions to detect the changes in the expression of the genes SOCS1 and bta-miR-30b-5p. Firstly, BoMac was treated with different concentrations (20, 50, 100, 200 ng/mL) of IFN-τ for 12 h, after which qRT-PCR was carried out. The results demonstrated that the expression of SOCS1 was gradually increased in a concentration-dependent manner, with the increase (*p* < 0.01) observed at 200 ng/mL. In contrast, the expression of bta-miR-30b-5p mRNA exhibited an inverse trend, with a decline (*p* < 0.05) in endogenous miR-30b-5p expression in macrophages following the administration of 200 ng/mL IFN-τ for 12 h (Figure 2A). Subsequently, stimulation at a concentration of 200 ng/mL for varying time periods demonstrated that SOCS1 mRNA expression reached a maximum at 12 h (*p* < 0.01) and subsequently declined (Figure 2B). Conversely, bta-miR-30b-5p mRNA expression reached a minimum at 12 h (*p* < 0.01), exhibiting a time-dependent pattern. Additionally, the expression of immunosuppressive factors was observed in IFN-τ-stimulated BoMac (Figure 2C). Immunofluorescence staining also demonstrated that the expression of SOCS1 and CD206 was most pronounced at the 12-h mark of 200 ng/mL IFN-τ stimulation (Figure 2D). Accordingly, 200 ng/mL IFN-τ acting on BoMac for 12 h was selected as the concentration and time point for subsequent experiments. CCK-8 assays demonstrated that the 200 ng/mL IFN-τ concentration employed in the experiments had no impact on cell survival (Figure 2E).

### 3.4. Effect of IFN-τ on SOCS1 and Downstream Pathway NF-κB Signaling Pathway

SOCS1 has the capacity to bind to the p65 subunit of the NF-κB pathway, thereby inducing its degradation. This, in turn, regulates a number of immune responses. To gain insight into the mechanisms underlying the observed macrophage polarization and the increase in immune factors, NF-κB p-65 and P-p65 protein expression in BoMac were investigated. The results are presented in Figure 2F,G. Following the administration of 200 ng/mL IFN-τ to BoMac for 12 h, a reduction (*p* < 0.05) in p-65 and P-p65 protein expression was observed, indicative of an inhibition in the NF-κB signaling pathway. In conclusion, these findings suggest that the downregulation of bta-miR-30b-5p may play a pivotal role in the polarization of macrophages toward M2 at the endometrium of dairy cows in early pregnancy by promoting SOCS1, which affects the NF-κB signaling pathway.

### 3.5. Bta-miR-30b-5p Has Been Demonstrated to Have a Targeting Relationship with SOCS1

**Figure 3:**
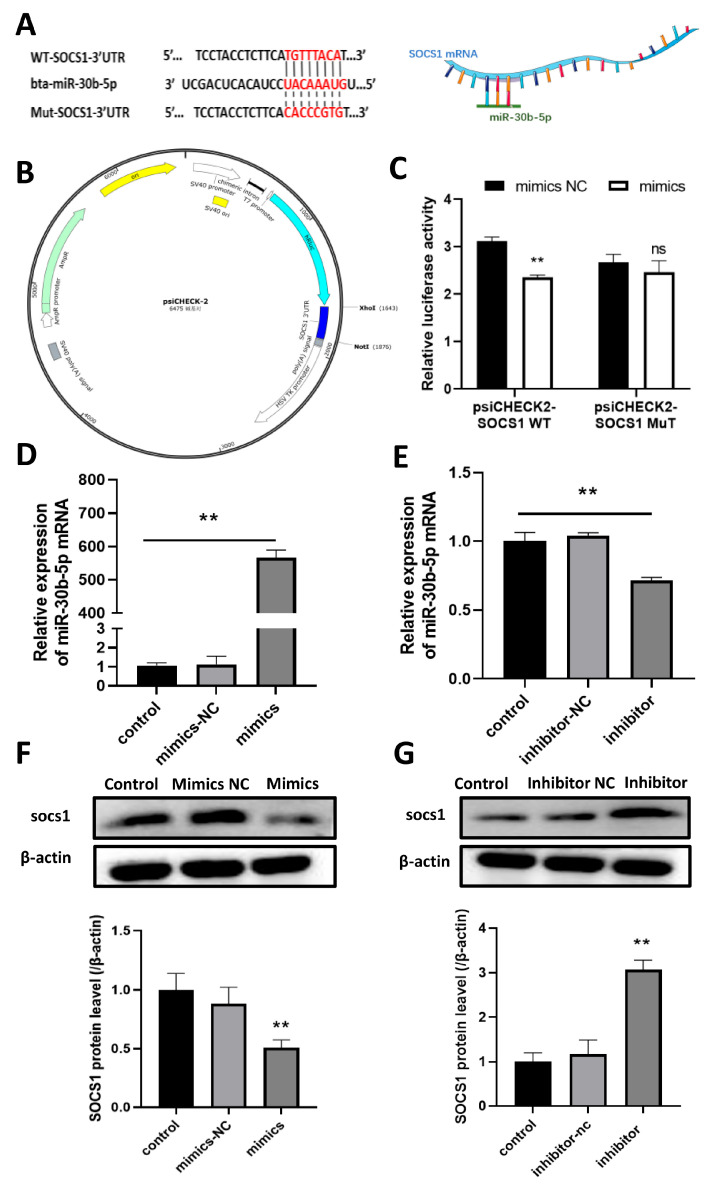
**SOCS1 is a direct target of bta-miR-30b-5p.** (**A**) Predicted binding site of SOCS1 3′UTR region to bta-miR-30b-5p. (**B**) Wild-type psiCHECK-2-SOCS1 3′-UTR and mutant psiCHECK -2-SOCS1 3′-UTR dual luciferase reporter plasmids. (**C**) The SOCS1 3′UTR-WT vector or the SOCS1 3′UTR-MUT vector was co-transfected with the bta-miR-30b-5p mimics or the negative control (NC) into 293 T cells, and the luciferase activity was subsequently measured. (**D**,**E**) BoMac cells were transfected with bta-miR-30b-5p mimics or inhibitors for 12 h. The transfection efficiency was quantified by quantitative reverse transcription polymerase chain reaction (qRT-PCR) to assess the expression of bta-miR-30b-5p. (**F**,**G**) The cells were treated as in (**D**,**E**), and a western blot was employed to detect SOCS1 protein expression. The data were derived from three independent experiments and are expressed as the mean ± SEM. ** *p* < 0.01 (Student’s *t*-test), ns: no significance.

The TargetScan online prediction database has indicated the presence of a target binding site for the bta-miR-30b-5p seed region within the 3′UTR of SOCS1 mRNA (Figure 3A). To further validate the target-binding relationship of bta-miR-30b-5p with SOCS1, we conducted dual luciferase experiments. The wild-type psiCHECK-2-SOCS1 3′-UTR and mutant psiCHECK-2-SOCS1 3′-UTR dual-luciferase reporter plasmids were initially constructed (Figure 3B), which were then co-transfected with bta-miR-30b-5p mimics or NC into 293 T cells. The results of the experiment demonstrated that the psiCHECK-2-WT and mimics groups exhibited a reduction (*p* < 0.01) in luciferase activity relative to the NC group, whereas the psiCHECK-2MUT and mimics groups demonstrated no discernible change (Figure 3C).

MiRNAs are capable of performing biological functions through the targeting of messenger RNAs (mRNAs), resulting in either the inhibition of translation or the degradation of the targeted mRNA. Thus, bta-miR-30b-5p mimics and inhibitors were separately transfected into BoMac, and the transfection efficiency was assessed via RT-qPCR 24 h post-transfection. The results demonstrated that transfection of mimics and inhibitors resulted in an increase (*p* < 0.01) and decrease (*p* < 0.01) in bta-miR-30b-5p expression in BoMac (Figure 3D,E). The alterations in SOCS1 expression in BoMac were subsequently investigated. The western blot results demonstrated that the overexpression of bta-miR-30b-5p in macrophages resulted in a reduction (*p* < 0.01) in SOCS1 protein expression, whereas the transfection of a bta-miR-30b-5p inhibitor led to an increase (*p* < 0.01) in SOCS1 protein expression (Figure 3F,G). In conclusion, these results indicate that bta-miR-30b-5p in BoMac directly targets SOCS1 and that a reduction in bta-miR-30b-5p levels results in an increase in SOCS1 expression.

### 3.6. Bta-miR-30b-5p Inhibits the NF-κB Pathway by Negatively Regulating SOCS1, Thereby Promoting Macrophage Polarization Towards M2 and the Release of Immunosuppressive Factors

**Figure 4:**
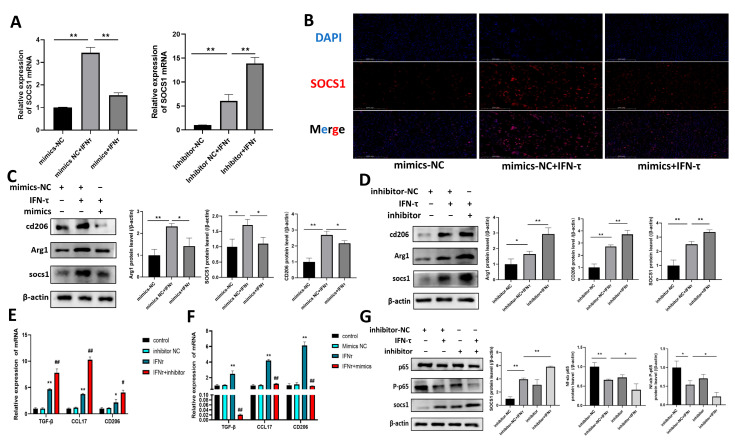
**Inhibit bta-miR-30b-5p, negatively regulate SOCS1, inhibit NF-κB signaling and promote M2 macrophage polarization** Initially, bta-miR-30b-5p mimics and inhibitors were transfected into BoMac, and then IFN-τ (200 ng/mL) was added for 12 h. (**A**) SOCS1 expression was detected by qRT-PCR. (**B**) Immunofluorescence images of SOCS1, with a scale bar measuring 500 μm. (**C**,**D**) Western blot analysis was employed to detect the protein expression of SOCS1 and M2 macrophage markers CD206 and Arg1. (**E**,**F**) The expression of immunosuppressive factors associated with the M2 phenotype was detected by qRT-PCR. The symbols * and # indicate, respectively, a comparison with the control group and a comparison with the IFN-τ group. (**G**) Detection of SOCS1 protein and p-65 and P-p65 protein expression in the NF-κB signaling pathway by western blot. * indicates comparison with the control group and # indicates comparison with the IFN-τ group. The data were derived from three independent experiments and are expressed as the mean ± SEM. * *p* < 0.005, ** *p* < 0.001, # *p* < 0.05, ## *p* < 0.01 (Student’s *t*-test).

MiRNAs have been demonstrated to regulate macrophage polarization and function. It can be reasonably inferred that the observed increase in M2 macrophages at the endometrium is a consequence of the dysregulation of bta-miR-30b-5p, which affects the expression of the gene SOCS1. To test this hypothesis, the following studies were conducted. Initially, the expression of bta-miR-30b-5p was modified by transfecting the BoMac cell model with bta-miR-30b-5p mimics and inhibitors. The results demonstrated that treatment with IFN-τ markedly elevated SOCS1 expression (*p* < 0.01) in comparison to the NC group. However, the overexpression of bta-miR-30b-5p was observed to markedly suppress the regulatory impact of IFN-τ on SOCS1 (*p* < 0.01). Conversely, the inhibition of bta-miR-30b-5p resulted in elevated SOCS1 expression (Figure 4A). The immunofluorescence results were consistent and demonstrated that bta-miR-30b-5p exerts a negative regulatory effect on SOCS1 (Figure 4B). Moreover, the phenotype of these macrophages was corroborated by western blot analysis. In comparison to the NC group, IFN-τ treatment resulted in an increase (*p* < 0.01) in the protein expression of M2 markers, including macrophage mannose receptor 1 (CD206) and arginase 1 (Arg1). However, the overexpression of bta-miR-30b-5p was observed to suppress the induction of IFN-τ on M2 macrophages (*p* < 0.05). Conversely, the inhibition of bta-miR-30b-5p resulted in elevated protein expression of CD206 and Arg1 (Figure 4C,D). Furthermore, it was observed that elevating bta-miR-30b-5p expression in IFN-τ-stimulated BoMac led to the suppression of CD206, CCL17, and TGF-β1 expression (*p* < 0.01). Conversely, the introduction of an inhibitor of bta-miR-30b-5p resulted in a renewed elevation of expression of these immunosuppressive factors (*p* < 0.01), effectively augmenting IFN-τ-induced M2 polarization (Figure 4E,F). In conclusion, these data indicate that the inhibition of bta-miR-30b-5p in IFN-τ-stimulated BoMac results in the high expression of SOCS1, which contributes to the polarization of macrophages into the M2 phenotype.

Prior research has demonstrated that SOCS1 plays a role in regulating immune responses by modulating the NF-κB signaling pathway. In light of these findings, we postulated that bta-miR-30b-5p may also influence macrophage polarization via the SOCS1/NF-κB pathway. Proteins were extracted from transfected cells and the protein expression of SOCS1, NF-κB p-65, and P-p65 was detected by western blot assay. The results demonstrated that inhibition of bta-miR-30b-5p was effective in increasing the expression of SOCS1 (*p* < 0.01) and decreasing the protein expression of p-65 and P-p65 (*p* < 0.05) (Figure 4G). In conclusion, the results demonstrated that bta-miR-30b-5p could inhibit the NF-κB pathway by negatively regulating SOCS1, thereby promoting macrophage polarization toward M2.

### 3.7. Silencing SOCS1 Prevents bta-miR-30b-5p-Regulated M2 Macrophage Polarization

**Figure 5:**
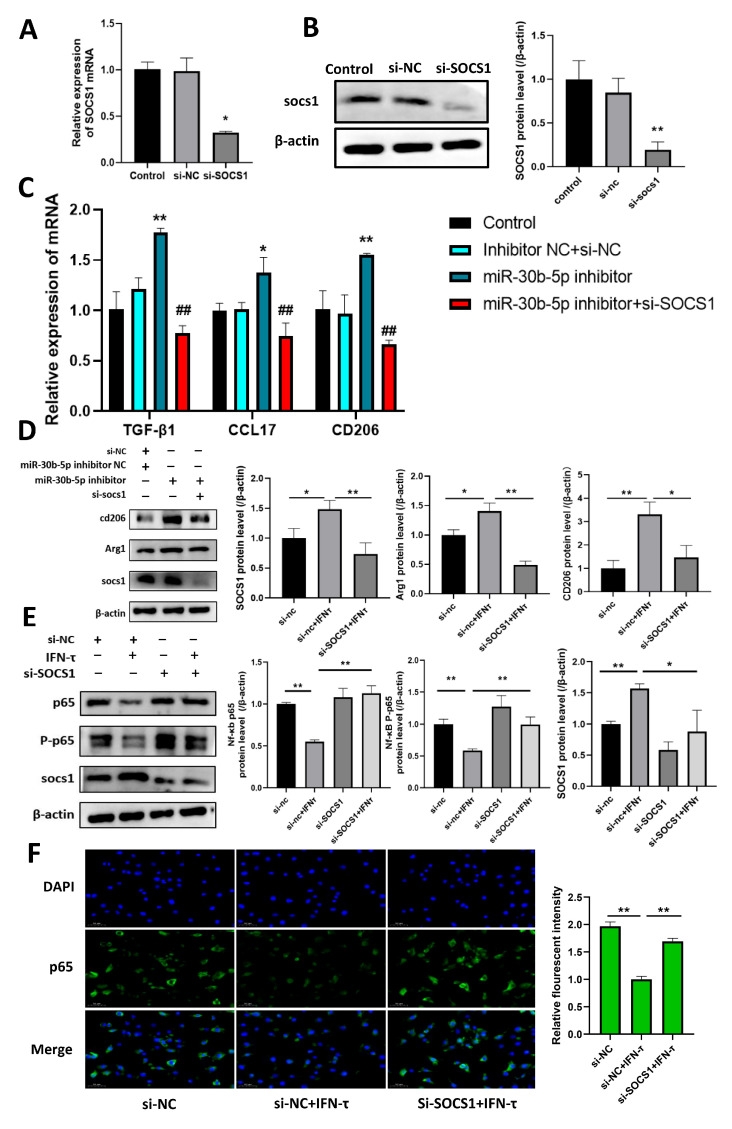
**Inhibiting SOCS1 expression enhances NF-κB signaling and prevents bta-miR-30b-5p promotion of M2 polarization** (**A**,**B**) The transfection efficiency was evaluated by transfecting si-SOCS1 into BoMac, and SOCS1 expression was quantified by qRT-PCR and western blot. (**C**) The transfection of bta-miR-30b-5p inhibitor or NC for 12 h in the presence or absence of si-SOCS1 was examined by qRT-PCR for the expression of M2 macrophage-associated immunosuppressive factors CD206, CCL17, and TGF-β1. (**D**) Cells were treated under the same conditions as in C, and protein expression of SOCS1, CD206, and Arg1 was detected by western blot. (**E**) BoMac was transfected with si-NC or si-SOCS1, followed by the addition or non-addition of IFN-τ, and p-65 and P-p65 protein expression in the NF-κB signaling pathway was detected by western blot. (**F**) Immunofluorescence was used to detect the p-65 expression level in the NF-κB signaling pathway. * indicates comparison with the control group and # indicates comparison with the inhibitor group. The data were derived from three independent experiments and are expressed as the mean ± SEM. * *p* < 0.005, ** *p* < 0.001, ## *p* < 0.01 (Student’s *t*-test).

We then investigated the role played by SOCS1 in bta-miR-30b-5p-regulated macrophage polarization by inhibiting the expression of this gene in BoMac through transfection of si-SOCS1. First, the results of qRT-PCR and western blot analysis demonstrated that the transfection of si-SOCS1 led to a reduction in the expression of SOCS1 in BoMac (*p* < 0.05) (Figure 5A,B). These findings are corroborated by the observation that the inhibition of bta-miR-30b-5p resulted in an increase (*p* < 0.01) in the expression of M2 markers, including CD206, CCL17, and TGF-β1. However, transfection of si-SOCS1 resulted in a reversal of this effect (*p* < 0.01) (Figure 5C). Western blot analysis further confirmed that silencing of SOCS1 resulted in decreased CD206 and Arg1 protein expression (*p* < 0.05) (Figure 5D). Furthermore, silencing SOCS1 resulted in a reversal of the protein expression of NF-κB p-65 and P-p65 (*p* < 0.01) (Figure 5E,F). Collectively, these data indicate that IFN-τ can act on macrophages aggregated at the endometrium during early pregnancy in dairy cows, promoting their polarization to the M2 type by inhibiting the endogenous bta-miR-30b-5p regulating the SOCS1/NF-κB signaling pathway.

### 3.8. Mechanism of IFN-τ on Macrophage Polarization at the Endometrium in Pregnant Mice

**Figure 6:**
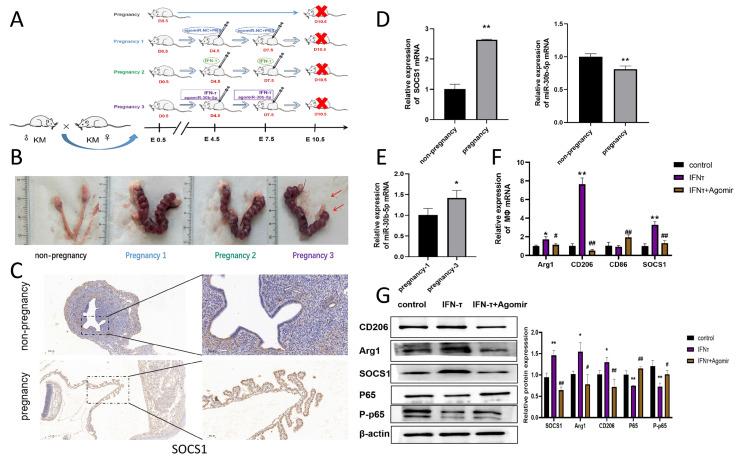
**Mechanism of action of IFN-τ on endometrial macrophage polarization in early pregnant mice** (**A**) Design of the experimental protocol for the mice. (**B**) Implantation of embryos in each group of mice. (**C**) Immunohistochemical images of SOCS1 in the endometrium of pregnant mice. (**D**) qRT-PCR analysis of SOCS1 and miR-30b-5p expression in the endometrium of mice in the pregnant group (*n* = 5) versus the nonpregnant group (*n* = 5). (**E**) qRT-PCR detection of no-microRNA-30b-5p injection following miR-30b-5p change. (**F**) Detection of SOCS1, M2 markers CD206 and Arg1, and M1 marker CD86 mRNA levels in the endometrium. (1, 2, 3). (**G**) Utilization of western blot to examine the pregnancy group (1, 2, 3) SOCS1, CD206, Arg1, P-p54, and p-65 protein levels. * indicates comparison with the control group and # indicates comparison with the IFN-τ group. The data were derived from three independent experiments and are expressed as the mean ± SEM. * *p* < 0.005, ** *p* < 0.001, # *p* < 0.05, ## *p* < 0.01 (Student’s *t*-test).

To further elucidate the regulatory mechanism of IFN-τ on endometrial macrophages in early pregnancy, a pregnant mouse model was established in this study. Pregnant mice were injected with either agomiR NC or IFN-τ, or a combination of IFN-τ and agomiR-30b-5p (Figure 6A). It is noteworthy that the number of embryos in pregnant mice was markedly diminished following the injection of agomiR-30b-5p in comparison to mice that received agomiR NC (Figure 6B). Immunohistochemical staining of the SOCS1 protein was conducted on mice in the non-pregnant and pregnant groups. The results, as illustrated in Figure 6C, demonstrated that SOCS1 exhibited high expression (*p* < 0.01) at the endometrium of pregnant mice in comparison to non-pregnant mice. qRT-PCR results were consistent with those observed in pregnant dairy cows, demonstrating an increase (*p* < 0.01) in SOCS1 expression within the endometrium of pregnant mice and a reduction (*p* < 0.01) in miR-30b-5p expression compared to non-pregnant mice (Figure 6D). However, miR-30b-5p expression was markedly elevated following agomiR-30b-5p treatment (*p* < 0.05) (Figure 6E). The detection of SOCS1 expression in the endometrium of mice in the pregnant group revealed an increase (*p* < 0.01) in SOCS1 expression in the IFN-τ group, whereas the addition of agomiR-30b-5p resulted in a decrease (*p* < 0.01) in SOCS1 expression. Furthermore, the increase in CD206 and Arg1 observed following IFN-τ stimulation was also reduced (*p* < 0.05) following the addition of agomiR-30b-5p (Figure 6F). Tissue proteins were extracted from the experimental samples, and the assay results demonstrated a decrease (*p* < 0.01) in NF-κB p-65 and P-p65 protein expression in the IFN-τ group. Conversely, this was reversed by the addition of agomiR-30b-5p (Figure 6G). Therefore, our findings indicate that miR-30b-5p may reduce M2 polarization by inhibiting SOCS1, which in turn activates the NF-κB signaling pathway, ultimately leading to embryo implantation failure. However, IFN-τ has been demonstrated to markedly improve this mechanism and maintain normal pregnancy status.

## 4. Discussion

IFN-τ, which is secreted by embryonic trophoblast ectodermal cells during the early stages of pregnancy in ruminants, is an important pregnancy cytokine that acts on the endometrium [32]. It has been reported that many of the immune response genes are under the control of IFN-τ [33]. The important role of IFN-τ in the immune response at the maternal–fetal interface has been confirmed by many researchers [34,35]. Macrophages are the most numerous population of immune cells during gestation in ruminants, and their specific functional phenotype influences the organism’s local immune homeostasis and tolerance [17,18]. However, there is a paucity of research regarding the mechanisms that regulate macrophage polarization in the context of bovine pregnancy. In this experiment, we observed a substantial increase in M2 macrophages in the uterine tissue of early bovine pregnancies. Therefore, we proposed that IFN-τ may modulate the immune status of pregnancy by regulating certain molecular mechanisms and influencing the polarization of macrophages. An increasing number of studies have demonstrated that SOCS1 is associated with a wide range of acute or chronic inflammatory diseases, as well as autoimmune disorders [36,37,38]. Furthermore, evidence suggests that its dysregulation can induce disease generation [39,40]. For example, mice lacking SOCS1 die within three weeks and show no signs of developing macrophage infiltration of major organs [41]. Mice lacking SOCS1 and CD28 develop SLE autoimmune disease [42]. Furthermore, evidence accumulated so far strongly suggests that SOCS1 demonstrates regulatory effects on a multitude of immune cells and modulates immune tolerance [43,44]. SOCS1 has been reported to limit IFN-γ responses in macrophages [38]. SOCS1 has been demonstrated to inhibit macrophage activation by inhibiting the JAK-STAT pathway and also affects the stability of NF-κB [45]. The SOCS1 protein plays a vital regulatory role in the regulation of various cytokines by innate immune cells [36]. Importantly, we examined the SOCS1 in bovine uterine tissue during early pregnancy and found increased expression. In light of these findings, we were prompted to investigate further whether IFN-τ regulates macrophage polarization through the SOCS1 gene, which in turn modulates the immune status in early pregnancy.

An increasing number of studies have demonstrated that miRNAs play a vital role in regulating biological processes [46,47]. It has a complex regulatory network with regard to cell proliferation, differentiation, apoptosis, inflammatory responses, and immune regulation [48,49]. For example, the upregulation of miR-33 in macrophages has been demonstrated to result in the elevation of pro-inflammatory cytokines and the M1 phenotype [50]. Overexpression of miR-34a in macrophages has been shown to favor a pro-inflammatory M1 phenotype and to inhibit lipopolysaccharide (LPS)-induced M2 polarization in mice with acute lung injury (ALI) [51]. Similarly, the overexpression of Let-7c and miR-99a miRNAs in mouse bone marrow-derived macrophages (BMDM) has been observed to reduce the angiotensin II-induced activation of the M1 type, while simultaneously promoting the M2 type [52]. Thus, when bta-miR-30b-5p was found to be significantly reduced in macrophages, it was reasonable to surmise that it was in a critical position. MiR-30b-5p is a member of the miR-30b family, which is highly conserved. Its dysregulation has been linked to a number of pregnancy-related illnesses, including preeclampsia and recurrent miscarriage [53]. Nevertheless, it remains uncertain whether the bta-miR-30b-5p reduction in M2 macrophages in cows during the early stages of pregnancy contributes to the immunomodulatory processes associated with pregnancy. Given the critical role of miRNAs in immune regulation, we were prompted to investigate the effect of bta-miR-30b-5p, which is declining in macrophages, on the immune status of pregnancy and to further explore whether it acts through SOCS1. As predicted, there is a target binding relationship between bta-miR-30b-5p and SOCS1. In vitro model of an IFN-τ-stimulated bovine macrophage cell, we observed a significant decrease of bta-miR-30b-5p. Furthermore, following the transfection of bta-miR-30b-5p into cells, we observed a reduction in SOCS1 expression, as well as a decrease in the secretion of immunosuppressive factors by macrophages, including TGF-β1, CCL17, and CD206. Western blot results similarly showed that transfection of bta-miR-30b-5p into BoMac, SOCS1 protein was reduced. It is noteworthy that the inhibition of bta-miR-30b-5p was found to markedly enhance M2 polarization, whereas the overexpression of bta-miR-30b-5p was observed to exert an opposing effect.

NF-κB signaling plays a role in a number of immune transcriptional processes, including the inflammatory response of innate immune cells to microbes and viruses, and the development and activation of adaptive immune cells [54,55]. It is strongly linked to immune and inflammatory responses. For example, interleukin 26 activates the NF-κB pathway to promote macrophage polarization towards M1 and ameliorate rheumatoid arthritis [56]. The triggering receptor for myeloid 2 (TREM2) expressed on the surface of mouse immune cells inhibits inflammation and modulates M2 macrophage polarization, ameliorating osteoarthritis pathology [57]. It can be concluded that the NF-κB pathway plays a crucial role in the process of macrophage polarization. In the IFN-τ-stimulated BoMac cell model, we examined the expression of the p-65 and P-p65 proteins within the NF-κB signaling pathway, which was found to be significantly reduced. It has been demonstrated that SOCS1 is capable of binding to the p65 subunit of NF-κB, thereby inducing its degradation [58]. Therefore, we speculate that SOCS1 may play an immunomodulatory role by regulating macrophage phenotype through inhibition of the NF-κB signaling pathway. In this study, we demonstrated that the inhibition of bta-miR-30b-5p led to increased SOCS1 expression and a reduction in protein levels of p-65 and P-p65 of NF-κB, thereby promoting the polarization of M2 macrophages. Conversely, the inhibition of SOCS1 expression led to an increase in p65 and P-p65 expression. The data illustrate that NF-κB signaling participates in the polarization of M2 macrophages, which is controlled by IFN-τ. It has been demonstrated that stimulation of IFN-τ during early pregnancy can regulate M2 macrophage polarization via the reduction of bta-miR-30b-5p, which acts on the SOCS1/NF-κB signaling pathway.

Although IFN-τ, secreted by embryonic trophoblast cells during pregnancy in ruminants, is species specific, IFN-τ exhibits sequence similarity and functional homology to IFNA in humans, mice, and rats [22]. Although IFN-τ is only produced in ruminants, it has been demonstrated to exhibit broad cross-species activity in humans and mice [59,60]. Furthermore, it has been shown to act in a variety of cell types, including macrophages, lymphocytes, and epithelial cells. For example, in animal models of multiple sclerosis, IFN-τ has been demonstrated to reduce fetal resorption and inhibit the development of experimental allergic encephalomyelitis (EAE), and to ameliorate spontaneous autoimmune diabetes in mice [61]. IFN-τ has been shown to attenuate lipopolysaccharide-induced inflammation by inhibiting the activation of the NF-κB and MAPKs pathways in mice [16]. In particular, IFN-τ has been demonstrated to exhibit low cytotoxicity and a low incidence of adverse effects, which suggests that it may have therapeutic potential as an alternative to other type I IFNs [62]. Therefore, in order to further explore whether the IFN-τ-regulated mechanism of immune tolerance in early pregnancy is species universal, this experiment included mice in addition to the bovine species. In the mouse experimental model, the injection of IFN-τ into pregnant mice resulted in a reduction in miR-30b-5p expression, an increase in SOCS1 expression, and an increase in M2 macrophages in their endometrium. Conversely, overexpression of miR-30b-5p resulted in the inhibition of SOCS1 expression, the activation of the NF-κB signaling pathway, the inhibition of M2 polarization, the destruction of the immune-tolerant environment in early pregnancy, and the prevention of smooth embryo implantation. These findings are in concordance with the results observed in bovine experiments, thereby substantiating the extensive regulatory function of IFN-τ. Consequently, IFN-τ can be identified as a crucial mediator in the regulation of immune tolerance during the early stages of pregnancy, and bta-miR-30b-5p may emerge as a promising novel therapeutic target.

## 5. Conclusions

To summarize, our data demonstrated that IFN-τ promotes macrophage polarization towards M2 in early pregnancy and then enhances immune tolerance in the uterus and the; the mechanism affects the expression of bta-miR-30b-5p. Furthermore, we also found that bta-miR-30b-5p can regulate the NF-κB pathway by targeting SOCS1 in macrophages. Our discovery offers novel insight into the immune regulation of IFN-τ in early pregnancy and elucidates the mechanism by which bta-miR-30b-5p controls the immune response in pregnancy.

## Figures and Tables

**Table 1:** Sequences for bta-miR-30b-5p and siRNA SOCS1.

Mimics	Sense: UGUAAACAUCCUACACUCAGCU Anti-Sense: CUGAGUGUAGGAUGUUUACAUU
Mimics NC	Sense: UUCUCCGAACGUGUCACGUTT Anti-sense:ACGUGACACGUUCGGAGAATT
Inhibitor	Sense: AGCUGAGUGUAGGAUGUUUACA
Inhibitor NC	Sense: CAGUACUUUUGUGUAGUACAA
Si-SOCS1	Sense: GACAGAGGAACUGCUUCUUTT Anti-sense: AAGAAGCAGUUCCUCUGUCTT
si-NC	Sense:UUCUCCGAACGUGUCACGUTT Anti-sense:ACGUGACACGUUCGGAGAATT

**Table 2:** Primers for RT-qPCR.

Gene	Sequence
RT-U6	AACGCTTCACGAATTTGCGT
U6-F	CTCGCTTCGGCAGCACA
U6-R	AACGCTTCACGAATTTGCGT
RT-bta-miR-30b-5p	CTCAACTGGTGTCGTGGAGTCGGCAATTCAGTTGAGAGCTGAGT
bta-miR-30b-5p-F	GCCGAGTGTAAACATCCTAC
bta-miR-30b-5p-R	CTCAACTGGTGTCGTGGA
bta-CD206-F	GTGATGGATCCCCTGTGTCA
bta-CD206-R	GTTGGCTCAGGTTTGGGAGT
bta-CCL17-F	CCATTCCCCAAAAGGTGCTG
bta-CCL17-R	GCGAGTCACCAGCACAATGG
bta-SOCS1-F	CTCGTACCTCCTACCTCTTCATGTT
bta-SOCS1-R	ACAGCAGAAAAATAAAGCCAGAGA
bta-TGF-β1	GCCTGCTGAGGCTCAAGTT
bta-TGF-β1	GCCGGAACTGAACCCGTTA
bta-GAPDH-F	AAGGTCGGAGTGAACGGATT
bta-GAPDH-R	ATGACGAGCTTCCCGTTCTC
mus-SOCS1-F	GTCCTGCCGCCAGATGAG
mus-SOCS1-R	GAGACAGAGGCAGTGAGCC
mus-CD206-F	TTCAGCTATTGGACGCGAGG
mus-CD206-R	GAATCTGACACCCAGCGGAA
mus-Arg1-F	AGCACTGAGGAAAGCTGGTC
mus-Arg1-R	TACGTCTCGCAAGCCAATGT
mus-CD86-F	ATGGACCCCAGATGCACCAT
mus-CD86-R	CGGCAGATATGCAGTCCCAT
mus-GAPDH-F	TGCACCACCAACTGCTTAG
mus-GAPDH-R	GGATGCAGGGATGATGTTC

## Data Availability

All other data supporting the results of this study are available upon request from the corresponding author. The RNA-seq dataset analyzed in this article is publicly available in the GEO database with the identifier https://www.oncotarget.com/article/18470/text/ (accessed on 21 December 2024).
